# STM visualisation of counterions and the effect of charges on self-assembled monolayers of macrocycles

**DOI:** 10.3762/bjnano.2.72

**Published:** 2011-10-11

**Authors:** Tibor Kudernac, Natalia Shabelina, Wael Mamdouh, Sigurd Höger, Steven De Feyter

**Affiliations:** 1 Department of Chemistry, Division of Molecular Imaging and Photonics, Laboratory of Photochemistry and Spectroscopy, Katholieke Universiteit Leuven, Celestijnenlaan 200 F, B-3001 Leuven, Belgium; 2 Molecular Nanofabrication Group, MESA+ Institute for Nanotechnology, University of Twente, P.O. Box 217, 7500AE Enschede, The Netherlands; 3 Kekulé-Institut für Organische Chemie und Biochemie, Rheinische Friedrich-Wilhelms-Universität Bonn, Gerhard-Domagk-Str. 1, 53121 Bonn, Germany; 4 Department of Chemistry, School of Sciences and Engineering, The American University in Cairo (AUC), AUC Avenue, P.O. Box 74, New Cairo 11835, Egypt

**Keywords:** counterions, liquid–solid interface, macrocycles, scanning tunnelling microscopy, self-assembly

## Abstract

Despite their importance in self-assembly processes, the influence of charged counterions on the geometry of self-assembled organic monolayers and their direct localisation within the monolayers has been given little attention. Recently, various examples of self-assembled monolayers composed of charged molecules on surfaces have been reported, but no effort has been made to prove the presence of counterions within the monolayer. Here we show that visualisation and exact localisation of counterions within self-assembled monolayers can be achieved with scanning tunnelling microscopy (STM). The presence of charges on the studied shape-persistent macrocycles is shown to have a profound effect on the self-assembly process at the liquid–solid interface. Furthermore, preferential adsorption was observed for the uncharged analogue of the macrocycle on a surface.

## Introduction

Ordered monolayers formed by the self-assembly of molecular building blocks on solid surfaces have recently attracted considerable attention [1–4], due to their promising use as functional surfaces in nanotechnological applications [5–6]. Recent successes in the rational design of self-assembled monolayers have opened new promising routes towards the controlled formation of regular molecular patterns with a wide range of symmetries and periodicities [1–4], with scanning tunnelling microscopy (STM) [7] being a primary characterisation tool. However, combining the ordering of self-organised, physisorbed monolayers with an additional functionality remains an important challenge. In principle, an additional functionality can be introduced in physisorbed molecular monolayers by co-adsorption of, for instance, thiols [8], combining physisorption and chemisorption. It can be envisioned that counterions of charged molecules that are adsorbed at the surface could be used not only to control the structure of 2D crystals at the liquid–solid interface, but also to control properties of the physisorbed molecules and to carry specific functionalities. For example, counterions have been shown to determine the chirality of self-assembled architectures [9]. Counterions also play a decisive role in supramolecular interactions and lead eventually to the formation of specific supramolecular complexes [10]. However, while counterions constitute an elegant way to modify electronic properties of molecules at the liquid–solid interface, their role in 2D self-assembly has been given little attention so far, mostly because they could not be visualised by STM [11–14].

**Figure 1:**
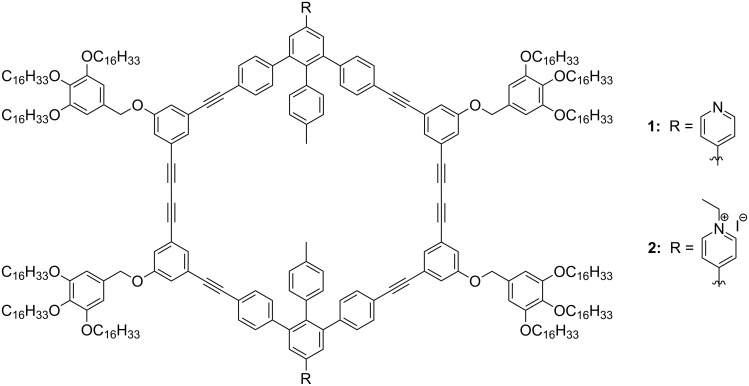
Structure of the shape-persistent macrocycles **1** and **2**.

Here, we report on self-assembly of a neutral shape-persistent macrocycle **1** and its charged analogue **2** (Figure 1) at the interface between an organic solvent and highly oriented pyrolytic graphite (HOPG). We demonstrate that the counterions of macrocycle **2** can be directly visualised by STM and that molecular packing is modified when the constitution of a monolayer is changed from uncharged to charged macrocycles. In macrocycle **2**, two identical positively charged centres are located on the nitrogen atoms, precisely on the rigid rim of the molecule. These positive charges are accompanied by two negatively charged iodide counterions.

## Results

### Self-assembly of neutral macrocycle **1**

**Figure 2:**
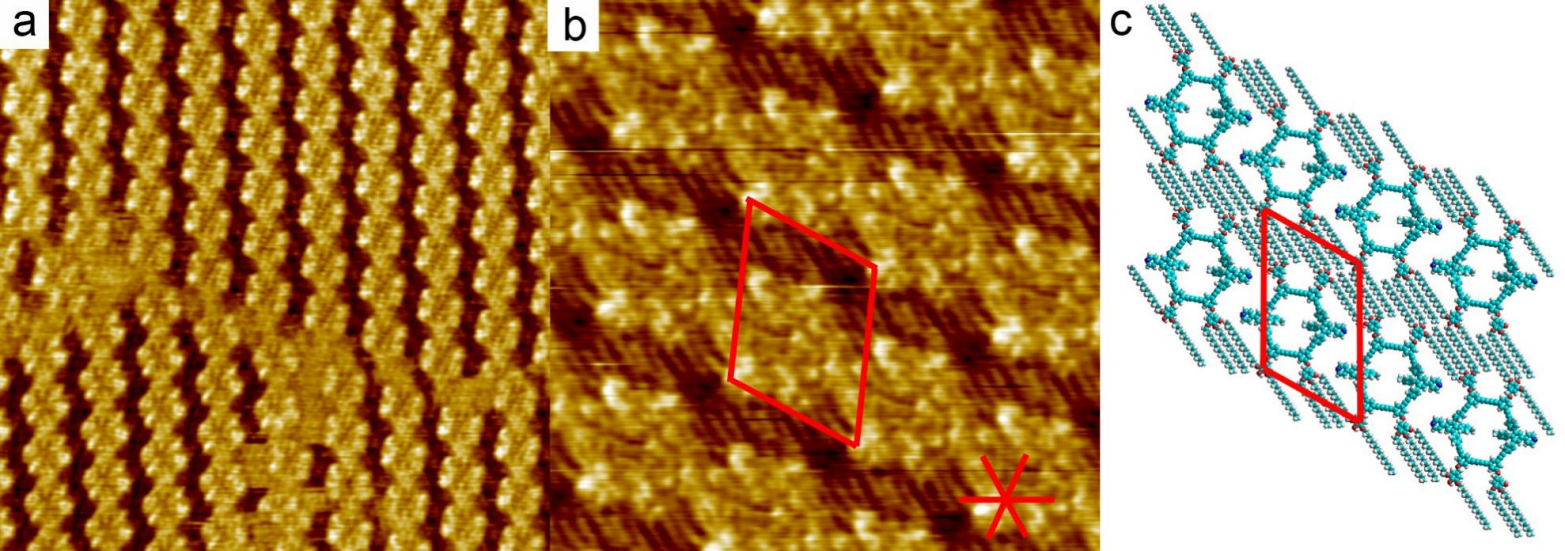
STM images and tentative molecular packing model of **1** self-assembled at the TCB/graphite interface. The unit cell parameters are *a* = 5.5 ± 0.1 nm, *b* = 4.4 ± 0.2 nm, γ = 68 ± 2°. a) 47.7 nm × 47.7 nm, *i*_T_ = 49 pA, *V*_S_ = −520 mV. b) High resolution STM image, 16.7 nm × 16.7 nm, *i*_T_ = 30 pA, *V*_S_ = −680 mV, the unit cell and the orientations of the main crystallographic directions of the underlying graphite surface are indicated in red. c) Schematic representation of the tentative packing model with the corresponding unit cell. Based on the STM contrast, on measurements of the area available for adsorption of alkyl chains, and on the ideal periodicity for physisorbed alkanes on graphite [18–19], it can be concluded that only eight out of twelve alkyl chains are adsorbed on graphite.

Highly ordered self-assembled monolayers of macrocycle **1** were formed by deposition of a 1,2,4-trichlorobenzene (TCB) solution of **1** onto a freshly cleaved HOPG surface. Figure 2a shows three different domains of **1**, separated by domain boundaries. Regularly spaced bright columns are tilted 30° away from the main crystallographic directions of the underlying graphite and comprise the aromatic parts of the molecule. High resolution images (Figure 2b) reveal that individual macrocycles are tilted either clockwise or counter-clockwise with respect to the direction of the alignment of molecular rows. Measurements of the area available for adsorption of alkyl side chains, together with the number of visualised alkyl chains (Figure 2b), suggest that one out of three chains is desorbed (dissolved in TCB). Figure 2c shows a tentative packing model for self-assembly of **1** at the TCB/HOPG interface. With respect to previously investigated shape-persistent macrocycles with similar shapes and sizes [15–17], domains formed by **1** show high crystallinity and order. Two types of symmetries of the self-assembled structures were previously realised. Low symmetry *p2* structures [15], comparable to the structures formed by **1**, and higher symmetry *pmm* structures [17]. The reasons for the differences in packing and symmetry cannot be easily rationalised. Consequently, guiding rules to achieving particular self-assembled structures are lacking.

### Co-assembly of charged macrocycle **2** and macrocycle **1**

**Figure 3:**
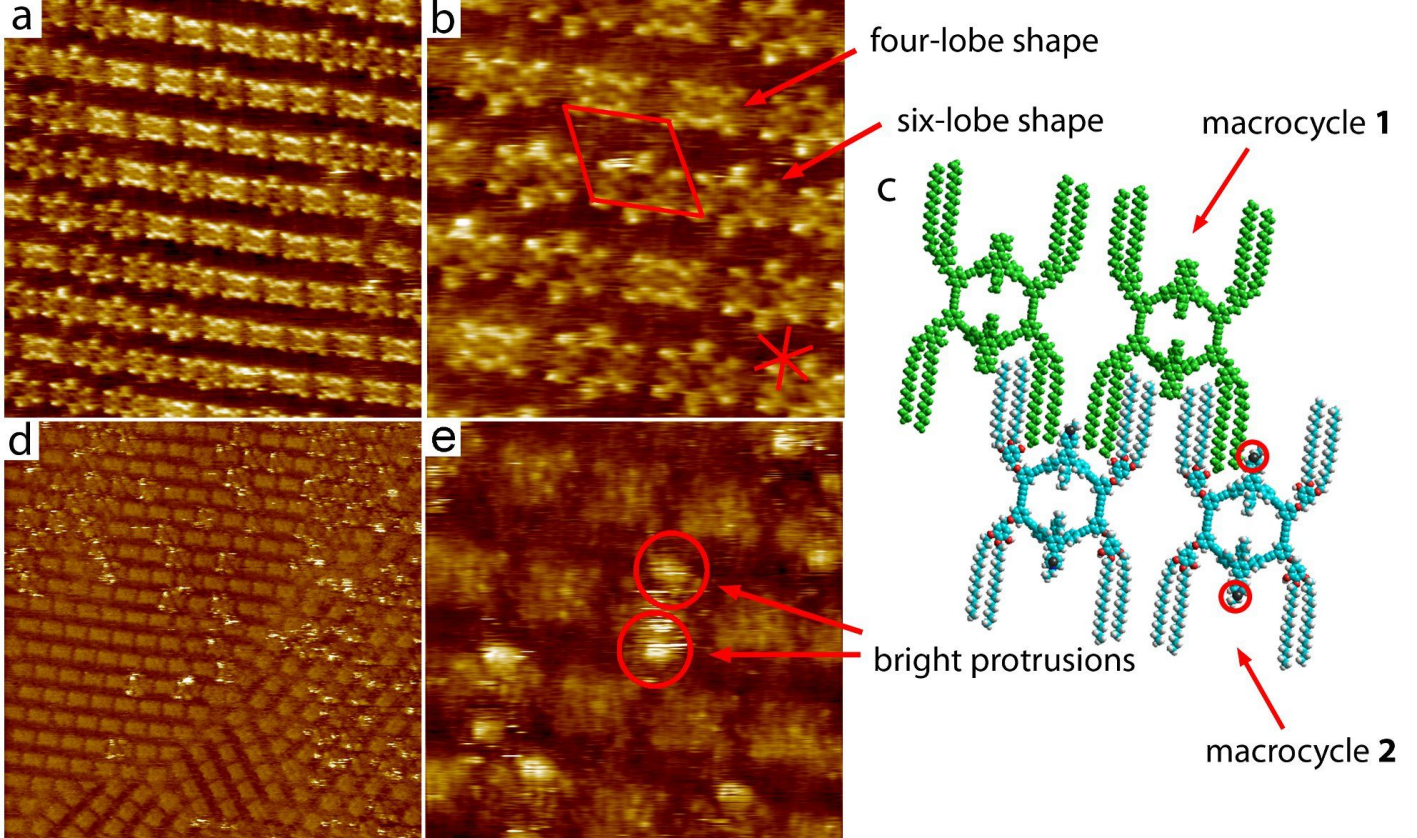
STM images and tentative molecular packing model of macrocycle **2** and **1** co-self-assembled at the trichlorobenzene/graphite interface. The unit cell parameters are *a* = 4.7 ± 0.2 nm, *b* = 4.4 ± 0.1 nm, γ = 65 ± 3°. a) 37.5 nm × 37.5 nm *i*_T_ = 67 pA, *V*_S_ = −554 mV. b) High resolution STM image, 17.2 nm × 17.2 nm, *i*_T_ = 45 pA, *V*_S_ = −554 mV, the unit cell and the orientations of the main crystallographic directions of the underlying graphite surface are indicated in red. c) Schematic representation of the tentative packing model of co-self-assembly of macrocycle **2** (bottom molecules) and **1** (top molecules in green). The iodide ions are depicted in black and highlighted by a red circle. Based on the STM contrasts, measurements of the area available for adsorption of alkyl chains, and the ideal periodicity for physisorbed alkanes on graphite [18–19], it can be concluded that only eight out of twelve alkyl chains per molecule are adsorbed on graphite. d) 67.8 nm × 67.8 nm, *i*_T_ = 36 pA, *V*_S_ = −537 mV, e) High resolution STM image, 18.1 nm × 18.1 nm, *i*_T_ = 37 pA, *V*_S_ = −561 mV.

Despite careful and repeated purification of the sample, attempts to investigate self-assembly of macrocycle **2** at TCB/HOPG interface always yielded co-assembled monolayers of **2** with what we believe is the uncharged analogue **1**, even when analytically pure samples of **2** were used (Figure 3). Various examples of co-assembly of shape-persistent macrocycles with additional molecules have been recently reported [17,20–27]. Note that **1** serves as a starting material for the synthesis of **2**. Figure 3a shows such typical STM image of a self-assembled monolayer containing a mixture of **2** and **1** at the TCB/HOPG interface. Scanning of larger areas reveals that the rims of the macrocycles present two different types of contrast (Figure 3a,b). One population of molecules is characterised by a four-lobe rectangular shape, whereas the molecules of the other population appear wider and with six-lobes (Figure 3b). The fact that these two macrocyclic objects show different tunnelling probabilities strongly suggests that they have different electronic structures. Consequently, based on the high resolution STM images (Figure 3b), we excluded the possibility that the two different contrasts that we observe arise from different orientations of the same molecule. Therefore, we conclude that the self-assembled monolayer is indeed formed by co-assembly of **1** and **2**. The presence of macrocycle **1** on the surface, while it is present in solution only in undetectable traces, clearly shows its preferential adsorption on HOPG (see discussion below) over the charged macrocycle **2**. However, co-assembly of **2** and **1** constitutes an ideal system to identify, unambiguously, the counterions by STM, where macrocycle **1** can be considered as an internal standard for the evaluation of different STM contrasts. The STM contrasts of both molecular species are distinct from the STM contrasts exhibited by the self-assembly of macrocycle **1** (Figure 2). This difference in the contrast is attributed to variations in the geometry (sharpness) of the STM tips used. Based on the high resolution images (Figure 3b) and the average area occupied by molecules, a tentative molecular packing model is proposed (Figure 3c). Molecules have eight alkyl chains adsorbed. The *pmm* symmetry (neglecting the chemical differences of **1** and **2**) of the self-assembled structures formed differs from the structures exclusively formed by macrocycle **1** (Figure 2).

When the scanning parameters were changed in such a way that the STM tip was slightly withdrawn from the surface (i.e., the current setpoint is decreased) the appearance of the molecules within the monolayer changed (Figure 3d). Two types of contrasts are again observed. One type of molecule gives sharp rectangular contrast, whereas the other type of molecule seems to be accompanied by protrusions with a very high contrast. Higher magnification STM images (Figure 3e) reveal that a pair of bright spots is always associated with one molecule. Isolated, individual bright spots were never observed in the course of these experiments. We anticipate that these bright protrusions correspond to the iodide counterions (see below).

### Assignment of STM contrasts to macrocycles and counterions

**Figure 4:**
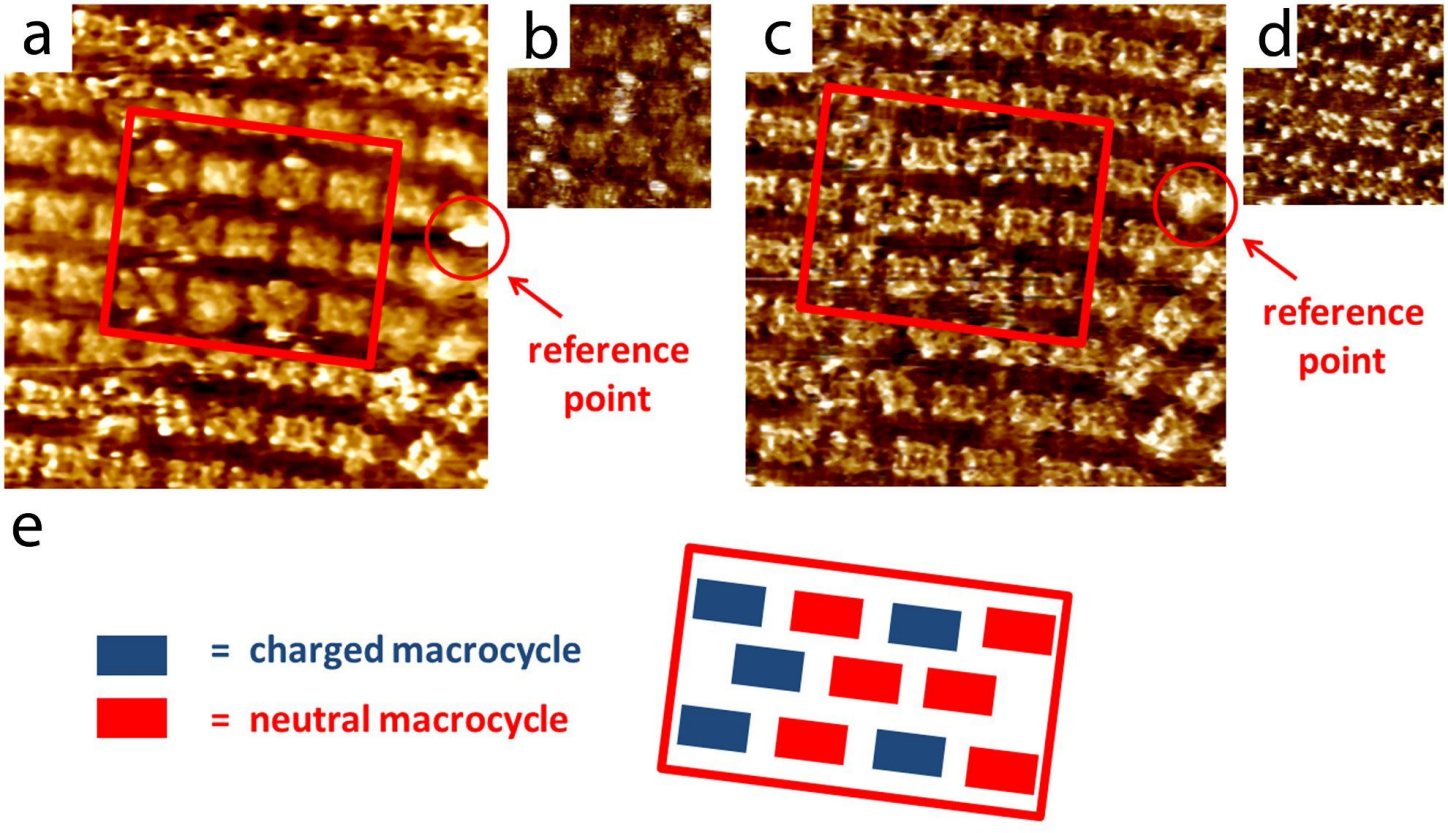
Assignment of STM contrasts to macrocycle **1** and **2**; a) 31 nm x 31 nm, *i*_T_ = 36 pA, *V*_S_ = −554 mV. STM images (a) and (c) were recorded at the same location on the HOPG surface. A bright spot highlighted by the red circle acts as a reference point. b) A typical high resolution STM image (18.1 nm × 18.1 nm, *i*_T_ = 37 pA, *V*_S_ = −561 mV) smoothened by 3-point median processing [30] in order to decrease the noise level and highlight the localisation of the iodide counterions. c) STM image of the same area as in Figure 4a scanned at higher tunnelling current, 29 nm x 29 nm, *i*_T_ = 67 pA, *V*_S_ = −554 mV. d) A typical high resolution STM image (17.2 nm × 17.2 nm, *i*_T_ = 45 pA, *V*_S_ = −554 mV) showing the internal structure of the macrocyclic backbone. e) Schematic representation of the composition of the scanned area highlighted in Figure 4a and 4c.

In order to assign, unambiguously, particular STM contrast to macrocycles **1** and **2** and to the iodide counterions, the same areas were scanned while scanning conditions were modified. For the duration of these experiments no dynamic exchange (desorption–readsorption) of molecules adsorbed on the surface was observed. Figure 4 shows again a typical STM image of a monolayer composed of both **2** and **1**. The area highlighted by the red rectangle (Figure 4a) shows eleven molecules with two different contrasts. In total five molecules, marked blue in the schematic representation in Figure 4e, show an STM contrast characterised by a pair of bright protrusions. This peculiar kind of contrast with high tunnelling current intensities at the “poles” of the molecules, is observed in other areas too (Figure 4b). The remaining six molecules, marked red in Figure 4e, appear flat and rectangular. When the STM tip–sample distance was reduced, the STM contrast of the molecules became sharper (Figure 4c). The apparent heights became similar for both populations, however, five “blue” molecules are considerably wider and two additional lobes on the top and bottom are still visible. Similar STM contrasts were observed in high resolution images from other areas (Figure 4d). By comparing the same molecules scanned under different conditions, we conclude that the species having the pairs of bright protrusions are associated with the species with the rims that appear wider. We intuitively assign the molecules with bright protrusions and wider rims to the charged macrocycle **2**. The bright protrusions, reflecting higher tunnelling probabilities, are thus associated with the iodide counterions. Iodides are expected to be located (averaged position in the timescale of the measurements) on top of the charged nitrogen atoms. As STM contrast is a convolution of the physical height and the local density of states [28], the large iodides “sitting” on top of the macrocycle rim logically result in a strong STM signal. Additionally, higher polarisability of iodine, as compared to the other atoms present, can contribute to higher tunnelling current [29]. This strategy demonstrates that the direct visualisation of counterions and their localisation on particular molecular species is possible by adjusting the tunnelling parameters.

## Discussion

Incorporation of charges, on macrocycle **2**, causes variation in the self-assembly of the macrocycle molecules. This is evidenced from the differences in the packing of pure **1** and mixed monolayers of **2** and **1**. Steric effects can be excluded as the shapes and sizes of **1** and **2** are effectively unaltered. The formation of the mixed monolayer suggests that the molecule–substrate interactions are considerably stronger for macrocycle **1** than for macrocycle **2**. No separation of **1** and **2** into different domains was observed. A tentative explanation for the different packing of pure **1** and the mixed monolayers of **1** and **2** is the tendency of the same charges, positive on the molecules and negative on the counterions, to be separated by the maximum possible distance while still preserving the high density of packing. The same tendency applies for the local interfacial dipoles that are formed upon adsorption of the molecule on the surface [31]. By comparing the packing models, it is obvious that the distance between the charged nitrogen atoms of the neighbouring macrocycles **2** in the co-assembly of **2** and **1** (Figure 3e) is larger than it would be if the same packing was adopted as in the case of the monolayers formed exclusively by macrocycle **1** (Figure 2c).

It is important to note that, from mixtures of molecules **1** and **2**, uncharged **1** exhibits preferential adsorption on graphite. The unfavourable adsorption of the charged macrocycle **2** might stem from the fact that the free movement of the counterions in the proximity of the nitrogen atoms partially shelters both sides of the unsaturated backbone and thus decreases the probability of effective adsorption on the surface. In addition, upon adsorption of **2** the freedom of movement of the counterions will be restricted to one side of the molecule, thus decreasing the entropy. Uncharged macrocycle **1** on the other hand does not bear any charges or counterions and we can safely assume that its interactions with graphite are stronger.

## Conclusion

In conclusion, we demonstrated that the direct STM visualisation of counterions is possible for charged molecules that are self-assembled at the liquid–solid interface. The presence of charges has a profound effect on the structure of these self-assembled monolayers. Direct localisation of counterions enables in situ exploration of counterion exchange. The charged macrocycles act as templates directing the adsorption of counterions. The affinity of the charged molecules for HOPG is lower than the uncharged analogues. In future, more complex counterions with additional functionalities will be introduced.

## Experimental

All STM experiments were carried out at 20–24 °C. Experiments were performed using a PicoSPM microscope (Agilent). Tips were mechanically cut from PtIr wire (80:20 alloy, diameter 0.25 mm). Prior to imaging, solutions of **1** and **2** in 1,2,4-trichlorobenzene (1.4 × 10^−4^ mol/L) were prepared, and for each experiment a drop of the appropriate solution was applied onto a freshly cleaved surface of HOPG (grade ZYB, Advanced Ceramics Inc., Cleveland, OH). The STM investigations were then performed at the liquid–solid interface within 1.5 h of the initial dropcasting of the solution. The HOPG lattice was recorded by lowering the bias immediately after obtaining images of the 2D structure. Images were corrected for drift effects using the HOPG lattice as calibration grid (Scanning Probe Image Processor (SPIP) 4.1.7 software (Image Metrology ApS)).

## Supporting Information

File 1 Experimental details of synthesis and characterisation of macrocycles **1** and **2**.
