# Supplementary material for: STM visualisation of counterions and the effect of charges on self-assembled monolayers of macrocycles
Source: Beilstein J Nanotechnol. 2011 Oct 11;2:674–80. doi: 10.3762/bjnano.2.72 (PMC3201620; doi:10.3762/bjnano.2.72)
Supplement: File 1 — Experimental details of synthesis and characterisation of macrocycles 1 and 2. [file Beilstein_J_Nanotechnol-02-674-s001.pdf]

Supporting Information

for

STM visualisation of counterions and the effect of charges on self-assembled monolayers of macrocycles

Tibor Kudernac[1,2], Natalia Shabelina[3], Wael Mamdouh[1,4], Sigurd Höger*[3] and Steven De Feyter*[1]

Address: Department of Chemistry, Division of Molecular Imaging and Photonics, Laboratory of Photochemistry and Spectroscopy, Katholieke Universiteit Leuven, Celestijnenlaan 200 F, B-3001 Leuven, Belgium; [2]Current address: Molecular Nanofabrication Group, MESA+ Institute for Nanotechnology, University of Twente, P.O. Box 217, 7500AE Enschede, The Netherlands; [3]Kekulé-Institut für Organische Chemie und Biochemie, Rheinische Friedrich-Wilhelms-Universität Bonn, Gerhard-Domagk-Str. 1, 53121 Bonn, Germany and [4]Current address: Department of Chemistry, School of Sciences and Engineering, The American University in Cairo (AUC), AUC Avenue, P.O. Box 74, New Cairo 11835, Egypt.

Email: Steven De Feyter - Steven.DeFeyter@chem.kuleuven.be; Sigurd Höger - hoeger@uni-bonn.de

* Corresponding author

# Experimental details of synthesis and characterisation of macrocycles 1 and 2.

Contents

1 General information S2

2 Synthesis S3

1 General information

Reagents were purchased at reagent grade from commercial sources and used without further purification. All air-sensitive reactions were carried out by means of standard Schlenk techniques under argon. Reaction solvents (THF, piperidine, dichloromethane) were dried, distilled, and stored under argon according to standard methods, workup solvents were either used in "p.a." quality or purified by distillation. Prior to characterization and further processing, all solids and oils were dried overnight at room temperature under vacuum. ^{1}H and ^{13}C NMR spectra were recorded on a Bruker DPX 400 (400.1 MHz for ^{1}H and 100.6 MHz for ^{13}C). Chemical shifts are given in parts per million (ppm) referenced to residual ^{1}H or ^{13}C signals in deuterated solvents. Mass spectra were measured on a Finnigan ThermoQuest MAT 95 XL (EI-MS), and a Bruker Daltonics autoflex TOF/TOF (MALDI-MS; matrix material: DCTB, no salts added). *m*/*z* peaks smaller than 10% (compared to the basis peak) are not reported. UV–vis absorption spectra were either recorded on a Shimadzu UV-2100 spectrophotometer using 10 mm quartz cuvettes or during GPC analysis (diode array detection). Fluorescence experiments were run on a Horiba Jobin Yvon FluoroMax-4 spectrofluorometer in all-transparent quartz cuvettes by monochromatic excitation at the indicated wavelength. All samples were prepared as a solution in chloroform. Melting points were determined by means of a Leica DMLB microscope with resistive heating socket controlled by a Leica LMW transformer and a Testo 925 digital thermometer. Thin layer chromatography was conducted on silica gel coated aluminium plates (Macherey–Nagel, Alugramm SIL G/UV_{254}, 0.25 mm coating with fluorescence indicator). Silica gel 60 M (Macherey–Nagel, 0.040–0.063 mm) was used as the stationary phase for column chromatography. Gel permeation chromatography (GPC) was performed in THF (HPLC grade, stabilised with 2.5 ppm BHT) at room temperature. GPC analyses were run on an Agilent Technologies system at a flow rate of 1 mL/min with an IsoPump G1310 A, a diode array UV detector (G1315B) and PSS columns (Polymer Standards Service, Mainz, Germany; 10^{2}, 10^{3}, and 10^{5} Å, 5 μ, 8 × 300 mm). All molecular weights were determined versus PS calibration (PS standards from PSS, Mainz, Germany).

2 Synthesis

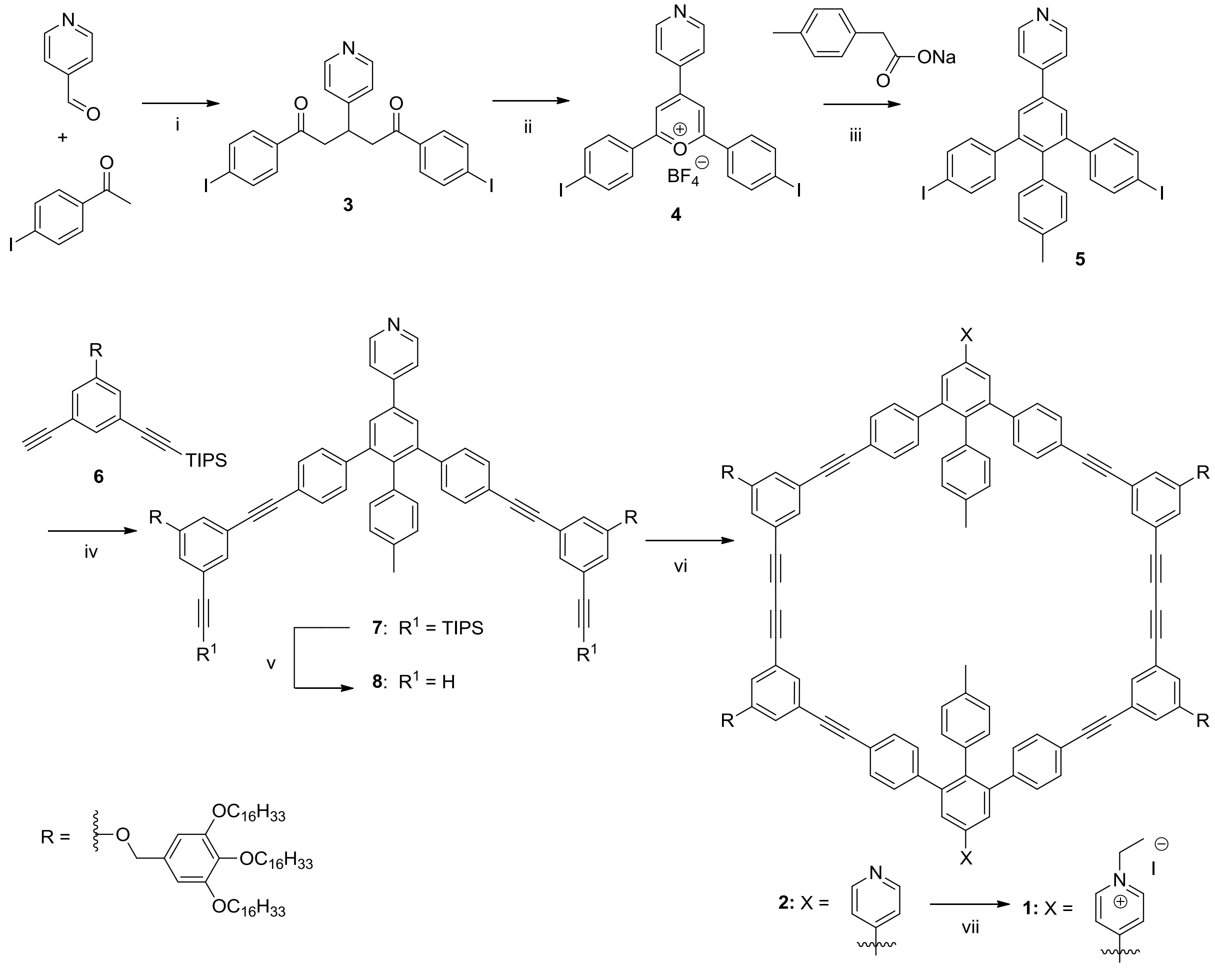

Scheme S1: (i) NaOH, MeOH, RT, 30%; (ii) $BF_3{\cdot}Et_2O$, chalcone, 2 h, 100 °C, 69%; (iii) Ac_2O, 3 h, 160 °C, 25%; (iv) $Pd(PPh_3)_2Cl_2$, PPh_3, CuI, piperidine, RT 52%; (v) Bu_4NF, THF, 2 h, RT, 70%; (vi) CuCl, $CuCl_2$, pyridine, 96 h, 40 °C, 63%; (vii) EtI, RT, 97%.

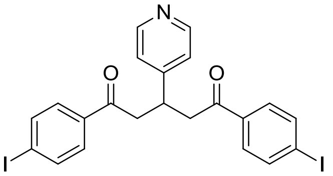

3

Synthesis of 3. Powdered NaOH (3.72 g, 93.00 mmol), 4-iodoacetophenone (22.97 g, 93.00 mmol), 4-pyridincarboxyaldehyde (4.00 g, 37.00 mmol) and MeOH (20 mL) were mixed together with a pestle and mortar for 30 min. The brown mixture was dissolved in CH_2Cl_2 and water, the organic phase was separated and washed with H_2O, brine and dried over $MgSO_4$, and the solvent was removed in vacuum. The residue was purified by column

chromatography over silica gel (CH_2Cl_2/ethyl acetate, 2:1, R_f: 0.27) and by recrystallisation from ethyl acetate to give **3** (30%, 6.53 g, 11.20 mmol) as a white powder; mp: 193–195 °C.

^{1}H NMR (400 MHz, ppm, CD_2Cl_2) δ 8.46 (d, *J* = 5.7 Hz, 2H), 7.84 (d, *J* = 8.58 Hz, 4H), 7.63 (d, *J* = 8.59 Hz, 4H), 7.21 (d, *J* = 6.02 Hz, 2H), 4.01–3.98 (m, 1H), 3.47–3.29 (m, 4H).

^{13}C NMR (100 MHz, ppm, $CDCl_3$) δ 196.83, 152.66, 149.93, 138.03, 135.70, 129.38, 122.89, 101.50, 43.58, 36.02.

MS EI *m/z* $(M)^+$: exact mass calculated for: ($C_{22}H_{17}I_2NO_2$): 580.9 g/mol, found: 580.9 g/mol.

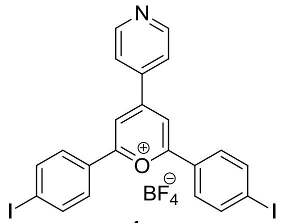

4

Synthesis of 4. Under an argon atmosphere, **3** (1.02 g, 1.76 mmol) and chalcone (0.415 g, 1.99 mmol) were dissolved in $BF_3 \cdot Et_2O$ (9 mL, 71.62 mmol) and stirred at 100 °C for 2 h. After cooling to RT, the brown precipitate was filtered off and washed with diethyl ether. After drying in vacuum **4** (69%, 0.79 g, 1.2 mmol) was obtained in the form of an orange powder.

^{1}H NMR (400 MHz, ppm, DMSO-d_6) δ 8.94 (d, *J* = 6.82 Hz, 2H), 8.30 (d, *J* = 6.82 Hz, 2H), 7.99–7.94 (m, 4H), 7.90 (s, 2H), 7.83 (d, *J* = 8.60 Hz, 2H), 7.78 (d, *J* = 8.54 Hz, 2H).

^{13}C NMR (100 MHz, ppm, DMSO-d_6) δ 195.23, 189.65, 137.49, 136.75, 136.31, 135.21, 130.06, 129.62, 128.06, 124.25.

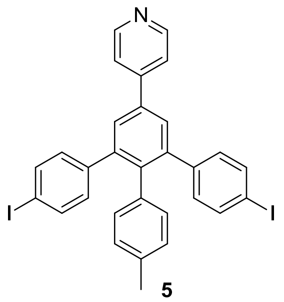

5

Synthesis of 5. 4 (3.00 g, 4.61 mmol) and sodium *p*-tolylacetate (prepared from *p*-tolylacetatic acid (6.24 g, 41.60 mmol), sodium hydroxide (3.33 g, 83.21 mmol) in MeOH/H_2O (44 mL; 10:1), followed by evaporation of the solvent) were suspended in acetic anhydride (2 mL) and refluxed at 160 °C for 3 h. After cooling to RT, the mixture was dissolved in CH_2Cl_2 and water, the organic phase was separated and washed with water, sodium hydroxide (10%), water, acetic acid (10%), water, brine, and dried over $MgSO_4$. After

evaporation of the solvent, the crude product was purified by column chromatography (CH_2Cl_2/ethyl acetate, 1:1, R_f: 0.50) and by recrystallisation from ethyl acetate to give, **5** (25%, 0.75 g, 1.14 mmol) as a white powder; mp: 211–213 °C.

^{1}H NMR (400 MHz, ppm, $CDCl_3$) δ 8.68 (d, J = 6.09 Hz, 2H), 7.63 (s, 2H), 7.57 (d, J = 6.14 Hz, 2H), 7.52 (d, J = 8.44 Hz, 4H), 6.86–6.83 (m, 6H), 6.70 (d, J = 8.05 Hz, 2H), 2.25 (s, 3H).

^{13}C NMR (100 MHz, ppm, $CDCl_3$) δ 142.00, 140.89, 139.74, 136.90, 136.32, 134.88, 131.62, 131.08, 128.53, 128.11, 92.56, 21.19.

MS EI m/z $(M)^+$: exact mass calculated for ($C_{30}H_{21}I_2N$): 648.9 g/mol, found: 649.0 g/mol.

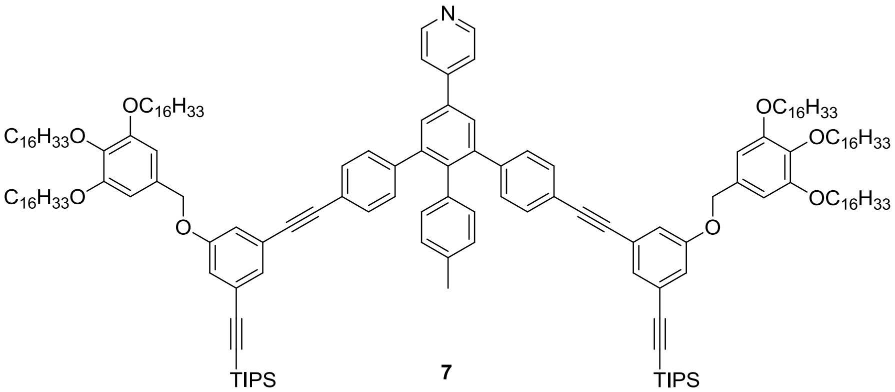

7

Synthesis of 7. (390 mg, 0.60 mmol), $Pd(PPh_3)_2Cl_2$ (120 mg, 0.17 mmol), PPh_3 (120 mg, 0.45 mmol), and CuI (60 mg, 0.30 mmol) were dissolved in piperidine (25 mL). A solution of **6** (1.44 g, 1.29 mmol) in THF (16 mL) was added and the mixture was heated to 40 °C over night. After cooling to RT, dichloromethane, diethyl ether and water were added; the organic phase was separated and washed with water, acetic acid (10%), water, sodium hydroxide (10%), water, and brine. The organic phase was dried over $MgSO_4$, filtered and evaporated in vacuum. The crude product was purified by column chromatography using dichloromethane as eluent (R_f: 0.25) to give **7** (52%, 818 mg, 0.31 mmol) as a white powder; mp: 48–50 °C.

^{1}H NMR (400 MHz, ppm, CD_2Cl_2) δ 8.67 (d, J = 5.86 Hz, 2H), 7.73 (s, 2H), 7.64 (d, J = 6.08 Hz, 2H), 7.36 (d, J = 8.32 Hz, 4H), 7.24–7.25 (m, 2H), 7.14 (d, J = 8.31 Hz, 4H), 7.09–7.08 (m, 2H), 7.06–7.05 (m, 2H), 6.99 (d, J = 8.00 Hz, 2H), 6.77 (d, J = 8.04 Hz, 2H), 6.62 (s, 4H), 4.96 (s, 4H), 3.98–3.90 (m, 12H), 2.23 (s, 3H), 1.81–1.70 (m, 12H), 1.48-1.42 (m, 12H), 1.25 (s, 144H), 1.10 (s, 42H), 0.89–0.85 (m, 18H).

^{13}C NMR (100 MHz, ppm, CD_2Cl_2) δ 158.36, 153.33, 150.02, 142.41, 141.69, 138.10, 136.22, 135.07, 131.24, 131.18, 131.12, 129.83, 128.45, 128.20, 128.09, 124.79, 124.34,

121.67, 121.10, 118.58, 117.90, 106.17, 91.34, 89.68, 88.92, 73.43, 70.64, 69.16, 31.92, 30.35, 29.71, 29.66, 29.50, 29.43, 29.36, 26.14, 26.11, 22.69, 18.65, 14.11, 11.28.

MS MALDI TOF (Matrix: DCTB) *m*/*z* $(M)^+$: exact mass calculated for ($C_{178}H_{275}NO_8Si_2$): 2611.1 g/mol, found: 2614.1 g/mol.

GPC (PS calibration, M_{peak} in g/mol): 4240.

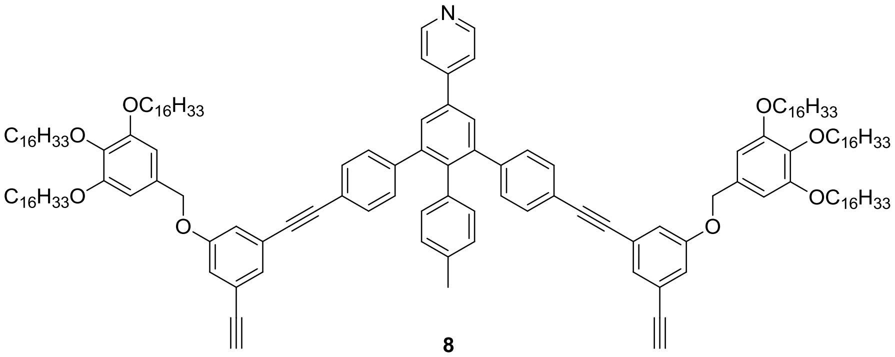

8

Synthesis of 8. A solution of Bu_4NF in THF (1 M; 0.03 mL, 0.03 mmol) was added under argon to a solution of **7** (33.2 mg, 0.012 mmol) in THF (1 mL). The mixture was stirred for 2 h at RT and then poured into diethyl ether and water. The organic layer was washed with water and brine, and dried over $MgSO_4$. The solvent was removed in vacuum and the residue was purified by column chromatography over silica gel with dichloromethane (R_f: 0.15) to give **8** (70%, 19 mg, 0.0084 mmol) as a white solid; mp: 49–50 °C.

^{1}H NMR (400 MHz, ppm, CD_2Cl_2) δ 8.68 (d, *J* = 4.41 Hz, 2H), 7.73 (s, 2H), 7.64 (d, *J* = 5.87 Hz, 2H), 7.36 (d, *J* = 8.46 Hz, 4H), 7.25–7.24 (m, 2H), 7.14 (d, *J* = 8.49 Hz, 4H), 7.12–7.11 (m, 2H), 7.07–7.06 (m, 2H), 6.88 (d, *J* = 8.28 Hz, 2H), 6.77 (d, *J* = 8.04 Hz, 2H), 6.61 (s, 4H), 4.95 (s, 4H), 3.98–3.91 (m, 12H), 3.14 (s, 2H), 2.23 (s, 3H), 1.80–1.69 (m, 12H), 1.48–1.44 (m, 12H), 1.26 (s, 144H), 0.88–0.85 (m, 18H).

^{13}C NMR (100 MHz, ppm, CD_2Cl_2) δ 158.20, 152.96, 149.81, 142.00, 141.73, 139.62, 137.50, 136.71, 136.07, 135.07, 130.96, 130.93, 130.66, 129.66, 127.93, 127.48, 124.23, 123.08, 120.55, 118.27, 118.12, 105.54, 89.37, 88.02, 82.11, 73.06, 70.30, 68.70, 31.61, 30.26, 29.43, 29.40, 29.34, 29.30, 29.11, 29.04, 25.83, 25.80, 22.37, 13.55.

MS MALDI TOF (Matrix: DCTB) *m*/*z* $(M)^+$: exact mass calculated for ($C_{160}H_{235}NO_8$): 2298.8 g/mol, found: 2300.8 g/mol.

GPC (PS calibration, M_{peak} in g/mol): 4025.

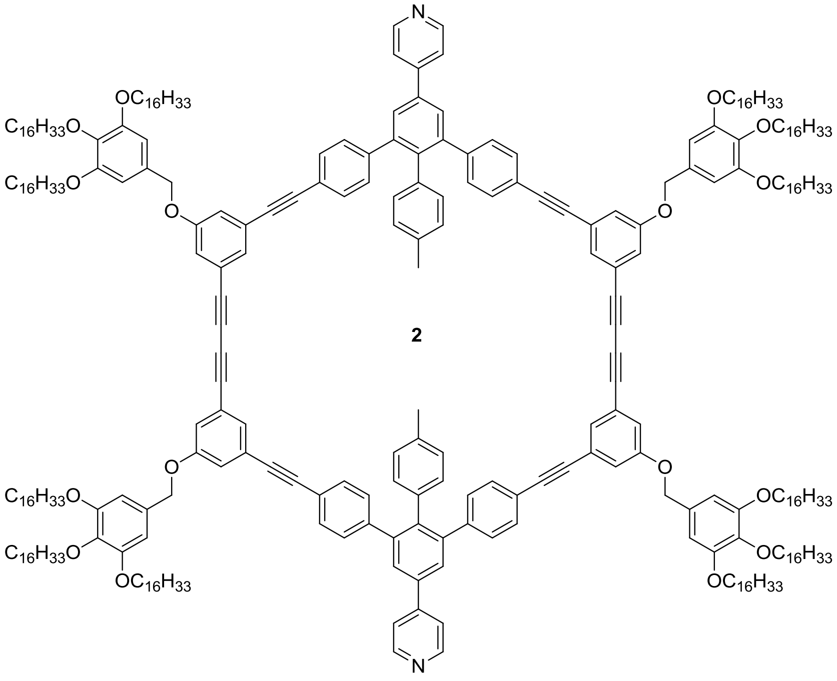

2

Synthesis of 1. Under an argon atmosphere a solution of **8** (111 mg, 0.048 mmol) in pyridine (35 mL) was added to a suspension of CuCl (957 mg, 9.66 mmol) and $CuCl_2$ (96 mg, 0.72 mmol) in pyridine (40 mL) over 96 h at 40 °C. After additional stirring for 24 h at RT, dichloromethane and water were added, and the organic phase was separated and washed with water, aqueous NH_3 solution (25%), water, acetic acid (10%), water, sodium hydroxide (10%), and brine. The organic phase was dried over $MgSO_4$ and concentrated to about 10 mL. Methanol was added and the precipitate was filtered off. The crude product was purified by radial chromatography (CH_2Cl_2/ethyl acetate 100:1, R_f: 0.20). After evaporation of the solvent and drying under vacuum, **1** (63%, 70 mg, 0.015 mmol) was obtained as white powder; mp: 99–100 °C.

^{1}H NMR (400 MHz, ppm, $CDCl_3$) δ 8.73 (d, *J* = 5.02 Hz, 4H), 7.85 (d, *J* = 4.03 Hz 4H), 7.77 (s, 4H), 7.36 (d, *J* = 7.73 Hz, 8H), 7.26 (s, 4H), 7.12–7.10 (m, 12H), 7.06–7.05 (m, 4H), 6.89 (d, *J* = 8.13 Hz, 4H), 6.71 (d, *J* = 8.04 Hz, 4H), 6.60 (s, 8H), 4.95 (s, 8H), 4.00–3.96 (m, 24H), 2.27 (s, 6H), 1.83–1.73 (m, 24H), 1.49–1.43 (m, 24H), 1.25 (s, 288H), 0.81–0.85 (m, 36H).

^{13}C NMR (100 MHz, ppm, $CDCl_3$) δ 158.55, 153.37, 142.76, 141.30, 138.20, 136.44, 135.64, 131.15, 131.09, 130.95, 129.82, 129.72, 129.69, 128.57, 128.21, 128.17, 124.70, 122.92, 122.51, 121.19, 118.52, 118.04, 106.17, 89.99, 88.55, 80.91, 73.45, 70.71, 69.19, 31.92, 30.35, 29.75, 29.71, 29.66, 29.63, 29.43, 29.41, 29.36, 26.14, 26.11, 22.69, 21.12, 14.11.

MS MALDI TOF (Matrix: DCTB) *m*/*z* $(M)^+$: exact mass calculated for ($C_{320}H_{466}N_2O_{16}$): 4593.5 g/mol, found: 4599.3 g/mol.

GPC (PS calibration, M_{peak} in g/mol): 7150.

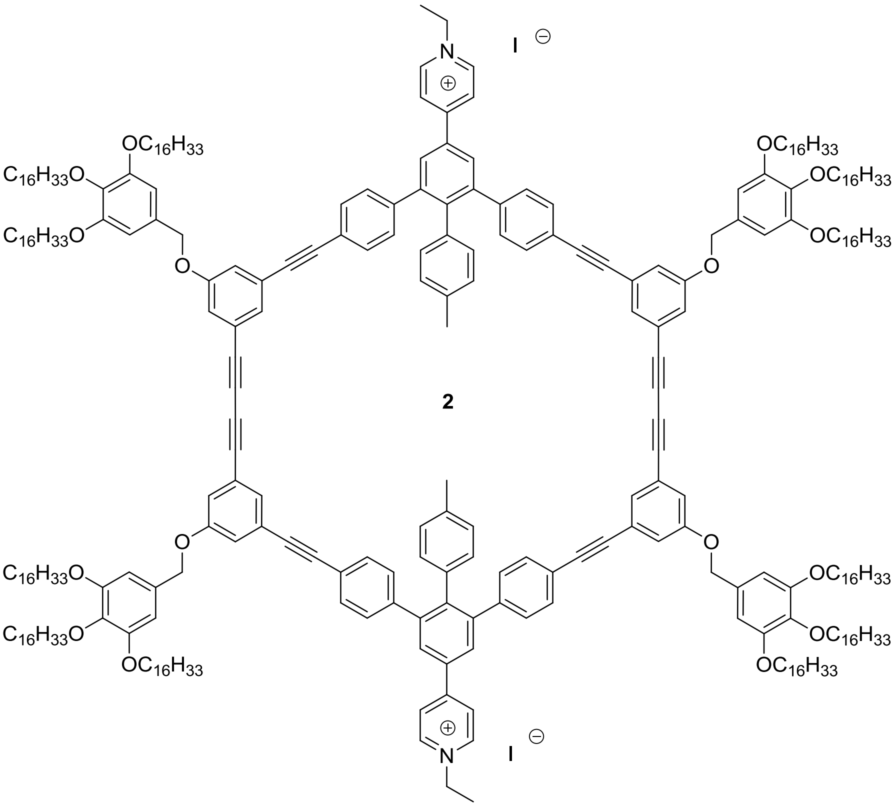

Synthesis of 2. Under an argon atmosphere, **1** (15 mg, 3.2 µmol) in EtI (3 mL) was stirred at RT over night. After evaporation of the solvent and drying under vacuum, **2** (97%, 15.3 mg, 3 µmol) was obtained as a slightly yellow powder (CH_2Cl_2/MeOH, 20:1, R_f: 0.22); mp: 315–320 °C (dec.).

^{1}H NMR (400 MHz, ppm, CD_2Cl_2) δ 9.25 (d, J = 6.18 Hz, 4H), 8.38 (d, J = 6.36 Hz, 4H), 7.89 (s, 4H), 7.37 (d, J = 8.01 Hz, 8H), 7.33 (s, 4H), 7.16–7.14 (m, 12H), 7.06 (s, 4H), 6.94 (d, J = 7.90 Hz, 4H), 6.75 (d, J = 7.79 Hz, 4H), 6.62 (s, 8H), 4.96–4.90 (m, 12H), 3.98–3.90 (m, 24H), 2.28 (s, 6H), 1.81–1.71 (m, 30H), 1.47–1.44 (m, 24H), 1.26 (s, 288H), 0.87–0.86 (m, 36H).

MS MALDI TOF (Matrix: DCTB) m/z $(M)^+$: exact mass calculated for $(C_{324}H_{474}N_2O_{16})^{2+}$: 2327.8 $[M]^{2+}$ g/mol, found: 2328.2 $[M]^{2+}$ g/mol.

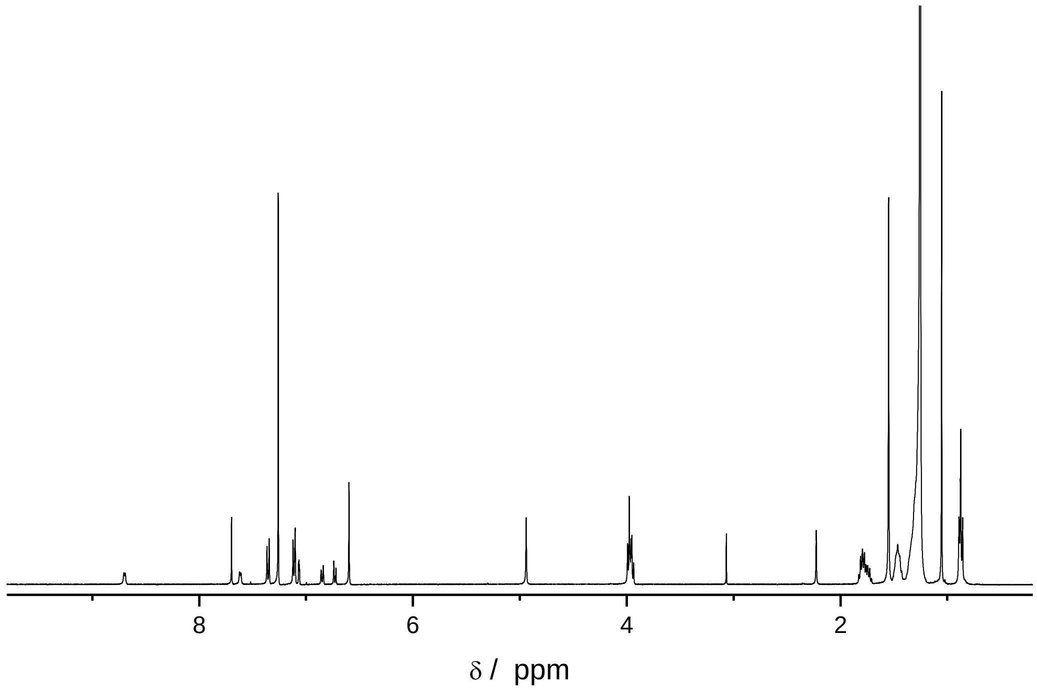

Figure S1: ^{1}H NMR ($CDCl_3$) spectrum of half ring **8**.

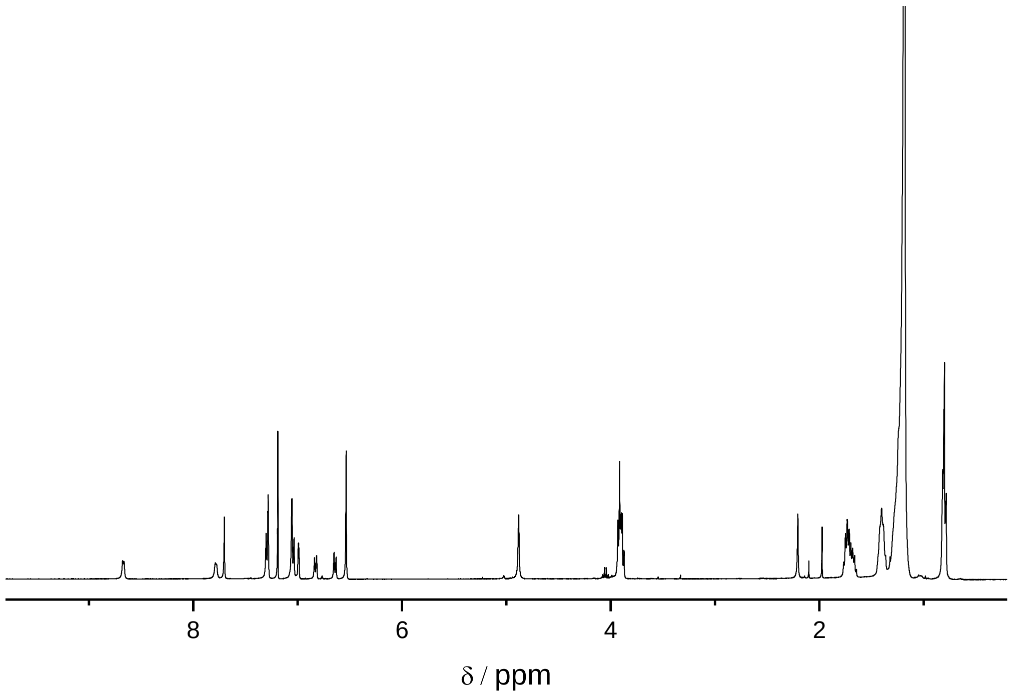

Figure S2: ^{1}H NMR ($CDCl_3$) spectrum of macrocycle **1**.

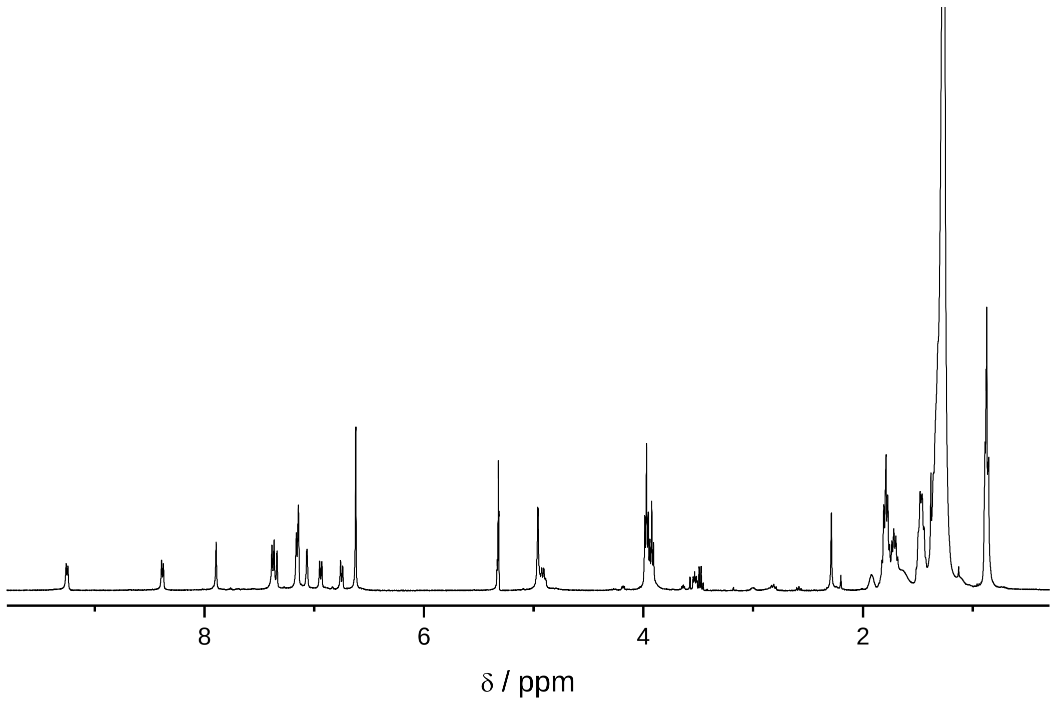

Figure S3: ^{1}H NMR (CD_2Cl_2) spectrum of cationic macrocycle **2**.
